# Genome-wide association mapping and gene expression analysis reveal candidate genes for grain chalkiness in rice

**DOI:** 10.3389/fpls.2023.1184276

**Published:** 2023-04-14

**Authors:** Xing Huo, Jian Wang, Luo Chen, Hua Fu, Tifeng Yang, Jingfang Dong, Yamei Ma, Lian Zhou, Jiansong Chen, Dilin Liu, Bin Liu, Junliang Zhao, Shaohong Zhang, Wu Yang

**Affiliations:** Rice Research Institute, Guangdong Academy of Agricultural Sciences, Guangdong Key Laboratory of New Technology in Rice Breeding, Guangdong Rice Engineering Laboratory, Key Laboratory of Genetics and Breeding of High Quality Rice in Southern China (Co-construction by Ministry and Province), Ministry of Agriculture and Rural Affairs, Guangzhou, China

**Keywords:** rice, grain chalkiness, genome-wide association study, quantitative trait loci (QTL), candidate gene

## Abstract

Grain chalkiness is the main factor determining the market value of rice. Reducing chalkiness is an important breeding goal for genetic improvement of high quality rice. Identification of QTLs or genes controlling chalkiness is the prerequisite for molecular breeding in rice. Here, we conducted a genome-wide association study to identify QTLs associated with grain chalkiness including percentage of grains with chalkiness (PGWC) and degree of endosperm chalkiness (DEC) in 450 rice accessions consisting of 300 *indica* and 150 *japonica* rice in two environments. A total of 34 QTLs were identified, including 14 QTLs for PGWC and 20 QTLs for DEC. Among them, seven QTLs were commonly identified in two environments, and eight QTLs were simultaneously related to two traits. Based on the haplotype analysis, LD decay analysis, RNA-sequencing, qRT-PCR confirmation and haplotype comparisons, four genes (*LOC_Os10g36170*, *LOC_Os10g36260*, *LOC_Os10g36340* and *LOC_Os10g36610*) were considered as the candidate genes for *qDEC-10c^1w,2wj^*, which could be identified in both environments and had the most significant *p*-value among the newly identified QTLs. These results provided new insight into the genetic basis of grain chalkiness and gene resources for improving quality by molecular breeding in rice.

## 1 Introduction

Rice is one of the most important food crops in the world. Improving rice yield and quality is the main goal of rice breeding. At present, with the improvement of people’s living standard, high quality rice is the primary concern of rice breeders and consumers. Rice quality mainly includes milling, appearance and cooking quality. Grain chalkiness, as an important character of appearance quality, is highly undesirable in rice breeding and marketing (Zhao et al., 2022). Percentage of grains with chalkiness (PGWC) and degree of endosperm chalkiness (DEC) are two indexes to evaluate grain chalkiness. Rice with high PGWC and DEC is easily broken during milling, and thus reducing the yield of head milled rice. Furthermore, the taste of chalky grain declines after cooking (Wu et al., 2022). Therefore, reducing chalkiness has been the key trait selected for released rice cultivars.

Understanding the genetic basis of grain chalkiness can greatly improve breeding efficiency. Grain PGWC and DEC are quantitatively inherited, controlled by multiple QTLs or genes, and affected by environments (Wang et al., 2017; Qiu et al., 2021). In past decade, bi-parental populations, rice mutants and reverse genetics have been widely used in QTL mapping and gene cloning for the two traits, and many QTLs have been reported and some genes have been cloned. *Chalk5* is cloned from natural variants and encodes a vacuolar H^+^-translocating pyrophosphatase. Elevated expression of *Chalk5* increases the endosperm chalkiness (Li et al., 2014). Recently, *WCR1* was identified by map-based cloning and up-regulated expression of *WCR1* significantly reduces white-core rate (Wu et al., 2022). Besides, some chalkiness-related genes are cloned from floury endosperm mutants, most of which are related to starch synthesis or starch quality. The *starch synthase IIIa* (*SSIIIa*) regulates short amylopectin elongation, and knockout of *SSIIIa* causes the formation of white core (Ryoo et al., 2007). The *GIF1* encodes a cell-wall invertase required for carbon partitioning during early grain-filling, and the *gif1* mutant shows loose starch grain structure and increased chalkiness (Wang et al., 2008). *OsLTPL36* is specially expressed in developing rice seed, and suppressed expression of this gene increases grain chalkiness rate (Wang et al., 2015). Fan et al. found that reduced expression of an autophagy gene *OsATG8b* results in chalky endosperm and poor seed quality (Fan et al., 2020).

In recent years, genome-wide association study (GWAS) based on linkage disequilibrium (LD) in a large germplasm resources has been widely used to identify QTLs that control complex traits, such as appearance quality in rice, on a large scale (Qiu et al., 2015; Wang et al., 2017; Qiu et al., 2021). Using a worldwide collection of *indica* rice germplasm, Qiu et al. identified two QTLs for PGWC and five QTLs for DEC, and two QTLs, *qPGWC5.1* and *qDEC5.2* on chromosome 5 were co-located with *GW5* (Qiu et al., 2015). Furthermore, they identified 12 QTLs for DEC in two years by using a larger panel mainly including *indica* and *japonica* from the 3K Rice Genomes Project, but none could be detected in both years (Qiu et al., 2021). Wang et al. detected four QTLs affecting DEC and four QTLs affecting PGWC, and haplotype analysis suggested that *Os07g0604500* was the candidate gene underlying *qDEC7* (Wang et al., 2017). Recently, *OsbZIP60* was identified as a vital regulator of grain chalkiness by GWAS, and knockout of *OsbZIP60* results in high grain chalkiness through regulating the expression of key genes related to grain chalkiness (Yang et al., 2022a). Although progress has been made in the identification and molecular mechanism of QTLs or genes associated with grain chalkiness in rice, but few of them have been applied to rice breeding. Therefore, it is necessary to further explore the stable QTLs or genes related to grain chalkiness in rice.

In the present study, PGWC and DEC in a diverse panel containing 450 accessions selected from the RDP2 (McCouch et al., 2016) were evaluated. GWAS was conducted to identify QTLs for the two traits. In total, 34 QTLs were identified including 11 QTLs co-localized with the reported QTLs or genes and 23 newly identified QTLs. Based on the linkage disequilibrium (LD) decay analysis, RNA-sequencing, qRT-PCR confirmation and haplotype comparisons, four genes were considered as candidate genes underlying *qDEC-10c^1w,2wj^*, which could be identified in both environments and had the most significant *p*-value among the newly identified QTLs. This study provides new insight into the genetic basis of grain chalkiness and contributes to molecular breeding for high-quality rice.

## 2 Materials and methods

### 2.1 Plant materials

The 450 rice accessions (**Table S1**) were used for phenotype evaluation and GWAS in this study. These rice accessions were selected from RDP2 (McCouch et al., 2016) based on their origins and diversity, including 300 *indica* and 150 *japonica* rice.

### 2.2 Phylogenetic analysis of 450 rice accessions

The 450 rice accessions from RDP2 had been genotyped by 700K SNPs (McCouch et al., 2016). The phylogenetic tree was constructed by SNPhylo using SNP data. The figure of phylogenetic tree was drawn by ITOL (Interaction Tree Of Life, https://itol.embl.de/).

### 2.3 Phenotypic evaluation of PGWC and DEC

The 450 rice accessions were planted in the experimental fields of Guangzhou (2016GZ, 23°8’ N and 113°17’ E) and Yangjiang (2018YJ, 21°89’ N and 111°90’ E) in Guangdong Province, China, in the second cropping season in 2016 and 2018, respectively. The experiments were performed in a randomized complete block design with two replicates in both environments. The field management, including irrigation, fertilization, and disease and pest control, followed the conventional or local practice in rice production. At complete maturity, the grains were harvested and dried naturally, and then stored in storage at 15 °C. 20 g grains were de-husked by a huller (JLG-III, Chengdu, China) and milled by a polisher (JNM, Chengdu, China). PGWC and DEC of head rice were detected by the Rice Appearance Quality Determination Instrument (SC-E, Hangzhou, Chian). PGWC was the percentage of grains with chalkiness, and DEC was calculated as the product of PGWC and chalkiness size (the area of chalkiness divided by the area of whole grain). All measurements were conducted using three independent samples from both two replicates, and the average values were used for subsequent GWAS analysis.

### 2.4 GWAS and QTL delimitation

GWAS was conducted as described in our previous study (Zhao et al., 2018; Yang et al., 2021) by using GAPIT version 2 software. Briefly, SNPs were filtered using the criteria of having less than 30% of missing data and minor allele frequency (MAF) > 0.05. The mixed linear model (MLM) with kinship matrix was used for GWAS, and the principal component was set to 3 in GAPIT. Manhattan and QQ plots were produced by R-package qqman (Turner, 2018). A region containing two or more than two significant SNPs (*p* < 0.0001) within 200 kb is considered as one QTL.

### 2.5 Linkage disequilibrium (LD) Decay analysis

The SNP information of about 900 kb (18.98-19.88 Mb) for *qDEC-10c^1w,2wj^* was used for LD Decay analysis. The LD heatmap were drawn by the R package LD heatmap.

### 2.6 RNA-Sequencing

Based on haplotype analysis and consistency of flowering time, six accessions with consistent flowering time, including three accessions with High DEC (accessions 787, 806 and 854) and three accessions with low DEC (accessions 730, 937 and 1046) were selected to conduct RNA-sequencing. The spikes were sampled on the 15th day after flowering, and total RNA was extracted using Trizol reagent (Takara, Dalian, China). RNA-sequencing was conducted by the Annoroad Gene Technology (Beijing, China), and data analysis was conducted as described in our previous study (Yang et al., 2022b). Genes with read count less than 30 were regarded as no expression. The differentially expressed genes between two sets of contrasting accessions were identified according to the criteria of *p*-value ≤ 0.01 and fold change of pairwise comparison ≥2 or ≤0.5.

### 2.7 DNA Re-Sequencing

The leaves of rice seedlings were collected and DNA extraction was conducted by CTAB method. The experiment was conducted according to the standard protocol provided by Illumina. The qualified genomic DNA was fragmented by ultrasonication. These fragments were then for purification, terminal repair and addition of A at the 3’ end, ligation and sequencing, and then fragmentation by agarosegel electrophoresis. Selection of appropriate DNA fragments and PCR amplification were performed to construct a sequencing library, and the library was sequenced using the Illumina NovaSeq6000 platform (BerryGenomics, Beijing, China). The raw sequence data have been reported in our previous study (Wang et al., 2023).

### 2.8 Real-time PCR analysis

The RNA samples for RNA-sequencing assays were used to confirm the expression level of candidate genes in this study. The cDNA synthesis was conducted using the PrimeScript^TM^ RT reagent kit (Takara, Japan). The qRT-PCR analysis was performed by qRT-PCR (Biorad CFX96, Pleasanton, CA, USA). The primers were designed by the Primer designing tool on NCBI. The gene-specific primers are listed in **Supplementary Table S5**. The *EF1α* was used as the normalized genes for mRNA. All reactions were repeated three times.

## 3 Results

### 3.1 Phenotypic variations of PGWC and DEC in 450 rice accessions

**Figure 1:**
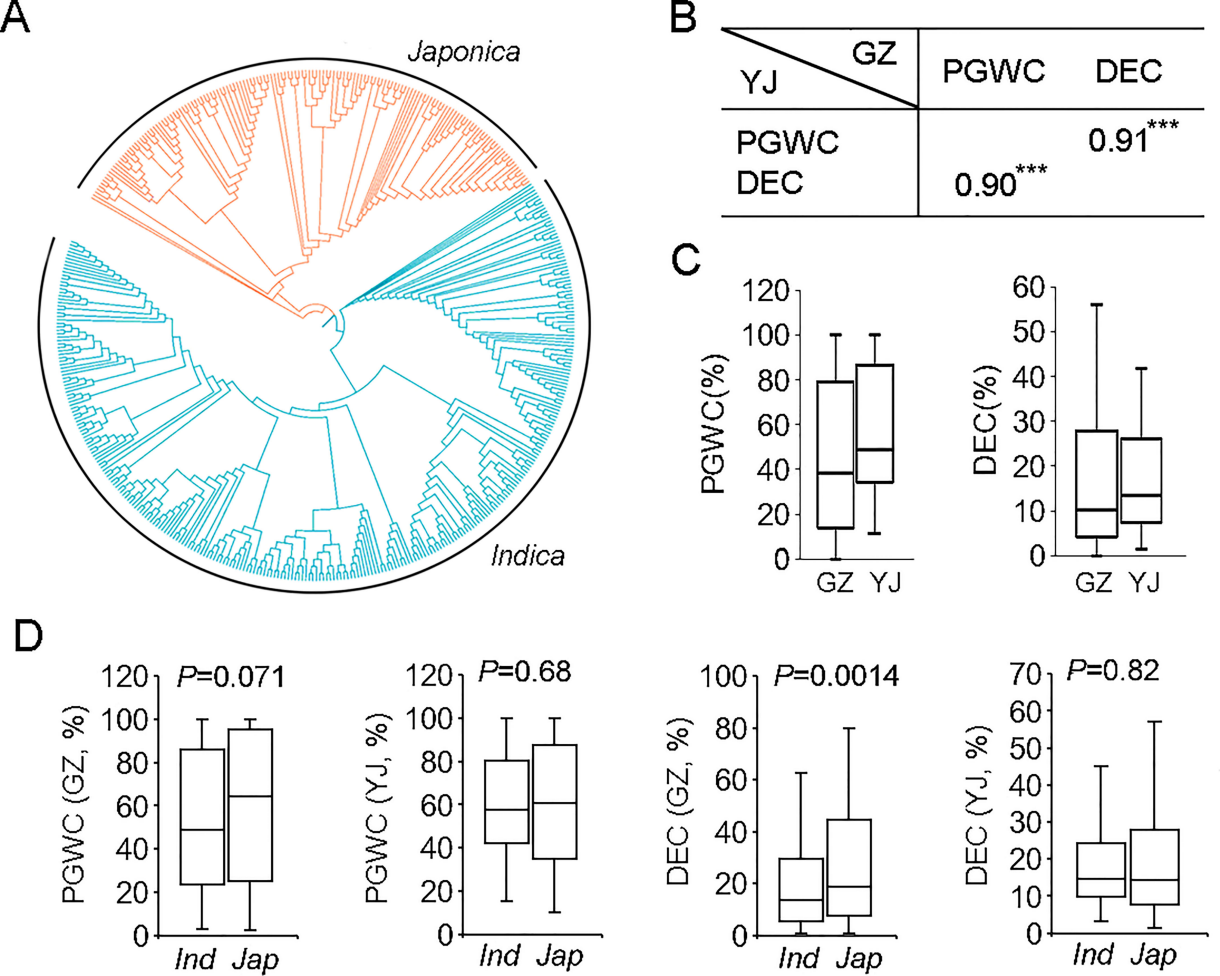
The phylogenetic tree and phenotypic comparison of 450 rice accessions used in this study. **(A)** The phylogenetic tree of 450 rice accessions. **(B)** Correlation analysis of PGWC and DEC in two environments. ***p<0.001. **(C)** Box plots of PGWC and DEC in two environments. **(D)** Phenotypic comparison between *indica* and *japonica* rice. Statistical comparison was performed by one-side *t*-test. GZ, Guangzhou; YJ, Yangjiang; PGWC, Percentage of grains with chalkiness; DEC, Degree of endosperm chalkiness.

Phylogenetic analysis based on their genotypes determined by the 700 K SNPs (McCouch et al., 2016) indicated that the 450 rice accessions could be roughly clustered into two groups, consisting of 300 *indica* and 150 *japonica* rice (**Table S1** and **Figure 1A**).

PGWC and DEC of the 450 rice accessions were evaluated in two environments (2016GZ and 2018YJ). The phenotype pairwise correlations between two traits were positively correlated in both environments, with correlation coefficients of 0.91 and 0.90 in 2016GZ and 2018YJ, respectively (**Figure 1B**). The panel showed a large variations for the two traits (**Figure 1C**). By comparing the phenotypes of *indica* and *japonica* rice, there were no significant differences between the two subpopulations in both environments (*p*>0.05) except that the DEC of *japonica* rice was significantly higher than that of *indica* rice in 2016GZ (*p*<0.01) (**Figure 1D**).

### 3.2 QTLs mapping for PGWC and DEC by GWAS

**Figure 2:**
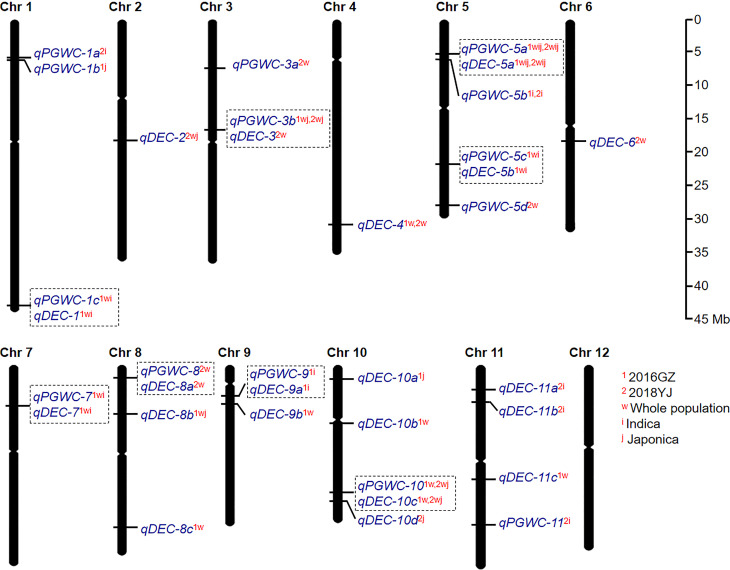
The global view of QTL mapping for PGWC and DEC by GWAS. The co-located QTLs were surrounded with dotted boxes. The superscript letters (1 and 2) refer to the two environments (2016GZ and 2018YJ, respectively). The superscript letters (w, i and j) refer to the three populations (whole, *indica* and *japonica*, respectively). For example, *qDEC-10c* ^1w,2wj^ indicates that *qDEC-10c* can be identified in 2016GZ by the whole population and in 2018YJ by the whole and *japonica* population.

A total of 34 QTLs (14 for PGWC and 20 for DEC) were identified in the two environments (**Figure 2**; **Figure S1** and **Table 1**). Among them, seven QTLs could be identified in both environments, while 15 and 12 QTLs were detected only in 2016GZ and 2018YJ, respectively. These QTLs were identified in different populations, including 11 QTLs in the whole population, 7 QTLs in the *indica* population, 3 QTLs in the *japonica* population, and 13 QTLs in more than one population.

**Table 1:** QTLs identified for PGWC and DEC in the present study.

QTL	Chromosome	Environment^†^	Population	The most significant SNP position (bp)^#^	*p*-value	Co-located QTL or cloned gene
PGWC						
*qPGWC-1a^2i^*	1	2018YJ	*Indica*	6,083,694	2.82E-05	*qDEC1* (Qiu et al., 2021)
*qPGWC-1b^1j^*	1	2016GZ	*Japonica*	6,241,628	3.47E-05	
*qPGWC-1c^1wi^*	1	2016GZ	whole	43,184,323	5.60E-05	
	1	2016GZ	*Indica*	43,241,738	2.94E-05	
*qPGWC-3a^2w^*	3	2018YJ	whole	7,795,320	1.97E-07	
*qPGWC-3b^1wj,2wj^*	3	2016GZ	whole	16,709,942	1.16E-06	*GS3* (Mao et al., 2010)
	3	2016GZ	*Japonica*	16,856,475	3.01E-06	
	3	2018YJ	whole	16,876,766	1.24E-07	
	3	2018YJ	*Japonica*	16,876,766	9.59E-06	
*qPGWC-5a* ^*1wij,2wij*^	5	2016GZ	whole	5,371,772	7.91E-16	*GW5* (Weng et al., 2008)
	5	2016GZ	*Indica*	5,359,520	5.18E-11	
	5	2016GZ	*Japonica*	5,342,339	7.41E-09	
	5	2018YJ	whole	5,371,772	4.60E-10	
	5	2018YJ	*Indica*	5,359,520	6.95E-06	
	5	2018YJ	*Japonica*	5,367,184	2.71E-06	
*qPGWC-5b^1i,2i^*	5	2016GZ	*Indica*	6,066,999	2.98E-05	
	5	2018YJ	*Indica*	6,005,763	2.11E-06	
*qPGWC-5c^1wi^*	5	2016GZ	whole	21,924,400	1.69E-05	
	5	2016GZ	*Indica*	21,944,065	1.65E-05	
*qPGWC-5d^2w^*	5	2018YJ	whole	27,961,292	4.14E-05	
*qPGWC-7^1wi^*	7	2016GZ	whole	6,211,855	2.99E-06	*qTr7.1* (Wang et al., 2017)
	7	2016GZ	*Indica*	6,181,910	3.47E-05	
*qPGWC-8^2w^*	8	2018YJ	whole	2,035,102	2.12E-05	
*qPGWC-9^1i^*	9	2016GZ	*Indica*	4,992,343	6.73E-06	
*qPGWC-10^1w,2wj^*	10	2016GZ	whole	19,480,113	4.84E-06	
	10	2018YJ	whole	19,481,956	2.41E-08	
	10	2018YJ	*Japonica*	19,481,956	1.30E-06	
*qPGWC-11^2i^*	11	2018YJ	*Indica*	24,399,195	2.01E-05	*qDEC11b* (Zhao et al., 2016); *qPGWC11c* (Zhao et al., 2016)
DEC						
*qDEC-1^1wi^*	1	2016GZ	whole	43,139,043	4.83E-05	
	1	2016GZ	*Indica*	43,118,717	1.81E-06	
*qDEC-2^2wj^*	2	2018YJ	whole	18,253,520	2.50E-05	
	2	2018YJ	*Japonica*	18,388,819	4.87E-05	
*qDEC-3^2w^*	3	2018YJ	whole	16,876,766	2.43E-06	*GS3* (Mao et al., 2010)
*qDCE-4^1w,2w^*	4	2016GZ	whole	30,708,157	1.54E-05	*PGC4.4* (Misra et al., 2021); *qWCR4* (Yun et al., 2016)
	4	2018YJ	whole	30,708,157	2.24E-05	
*qDEC-5a^1wij,2wij^*	5	2016GZ	whole	5,371,772	3.22E-12	*GW5* (Weng et al., 2008)
	5	2016GZ	*Indica*	5,359,520	8.12E-08	
	5	2016GZ	*Japonica*	5,359,740	4.50E-05	
	5	2018YJ	whole	5,419,124	1.23E-09	
	5	2018YJ	*Indica*	5,431,662	4.57E-06	
	5	2018YJ	*Japonica*	5,419,124	6.38E-06	
*qDEC-5b^1wi^*	5	2016GZ	whole	21,760,992	3.40E-05	
	5	2016GZ	*Indica*	21,873,455	1.10E-05	
*qDEC-6^2w^*	6	2018YJ	whole	18,799,852	2.91E-06	
*qDEC-7^1wi^*	7	2016GZ	whole	6,218,264	6.89E-10	*qTr7.1* (Wang et al., 2017)
	7	2016GZ	*Indica*	6,211,855	3.70E-08	
*qDEC-8a^2w^*	8	2018YJ	whole	2,035,102	6.91E-06	
*qDEC-8b^1wj^*	8	2016GZ	whole	7,796,155	1.04E-05	
	8	2016GZ	*Japonica*	7,754,397	7.17E-06	
*qDEC-8c^1w^*	8	2016GZ	whole	24,785,271	1.55E-05	*qWBA8-W+* (Peng et al., 2014); *qDEC8b* (Zhao et al., 2016); *qWBR8* (Yun et al., 2016)
*qDEC-9a^1i^*	9	2016GZ	*Indica*	4,962,744	2.86E-05	
*qDEC-9b^1w^*	9	2016GZ	whole	6,109,637	5.26E-06	*QPGWC.HN-9* (Bian et al., 2014)
*qDEC-10a^1j^*	10	2016GZ	*Japonica*	2,319,127	4.47E-05	
*qDEC-10b^1w^*	10	2016GZ	whole	9,320,097	3.97E-06	
*qDEC-10c^1w,2wj^*	10	2016GZ	whole	19,480,113	3.04E-05	
	10	2018YJ	whole	19,481,956	1.47E-10	
	10	2018YJ	*Japonica*	19,481,956	1.06E-08	
*qDEC-10d^2j^*	10	2018YJ	*Japonica*	20,500,122	2.25E-05	
*qDEC-11a^2i^*	11	2018YJ	*Indica*	3,950,541	1.13E-05	
*qDEC-11b^2i^*	11	2018YJ	*Indica*	5,770,569	7.68E-06	
*qDEC-11c^1w^*	11	2016GZ	whole	17,446,555	3.51E-05	

^†^ The experimental bases of Guangzhou in 2016 (2016GZ) and Yangjiang in 2018 (2018YJ).

^#^ Position of the most significant SNP at the QTL region.

Besides, it was found that some QTLs for PGWC and DEC were co-located on the same chromosomal regions. They were *qPGWC-1c* ^1wi^ and *qDEC-1* ^1wi^ on chromosome 1; *qPGWC-3b* ^1wj,2wj^ and *qDEC-3* ^2w^ on chromosome 3; *qPGWC-5a* ^1wij,2wij^ and *qDEC-5a* ^1wij,2wij^ on chromosome 5; *qPGWC-5c* ^1wi^ and *qDEC-5b* ^1wi^ on chromosome 5; *qPGWC-7* ^1wi^ and *qDEC-7* ^1wi^ on chromosome 7; *qPGWC-8* ^2w^ and *qDEC-8a* ^2w^ on chromosome 8; *qPGWC-9* ^1i^ and *qDEC-9a* ^1i^ on chromosome 9; *qPGWC-10* ^1w,2wj^ and *qDEC-10c* ^1w,2wj^ on chromosome 10. While the other 18 QTLs were associated with one trait (**Figure 2**).

Compared with the previous studies, 11 QTLs identified in this study were co-localized with the reported QTLs or cloned genes (**Table 1**). In particular, *qPGWC-3b* ^1wj,2wj^ and *qDEC-3* ^2w^ were co-located with *GS3*, a major gene regulating grain length (Mao et al., 2010); *qPGWC-5a* ^1wij,2wij^ and *qDEC-5a* ^1wij,2wij^ were co-located with *GW5*, a major gene regulating grain width (Weng et al., 2008) and several QTLs for chalky traits (Weng et al., 2008; Mao et al., 2010; Bian et al., 2014; Peng et al., 2014; Yun et al., 2016; Zhao et al., 2016; Wang et al., 2017; Misra et al., 2021; Qiu et al., 2021). The other 23 QTLs were newly identified in the present study.

### 3.3 Phenotype comparison and region analysis of *qDEC-10c^1w,2wj^*

**Figure 3:**
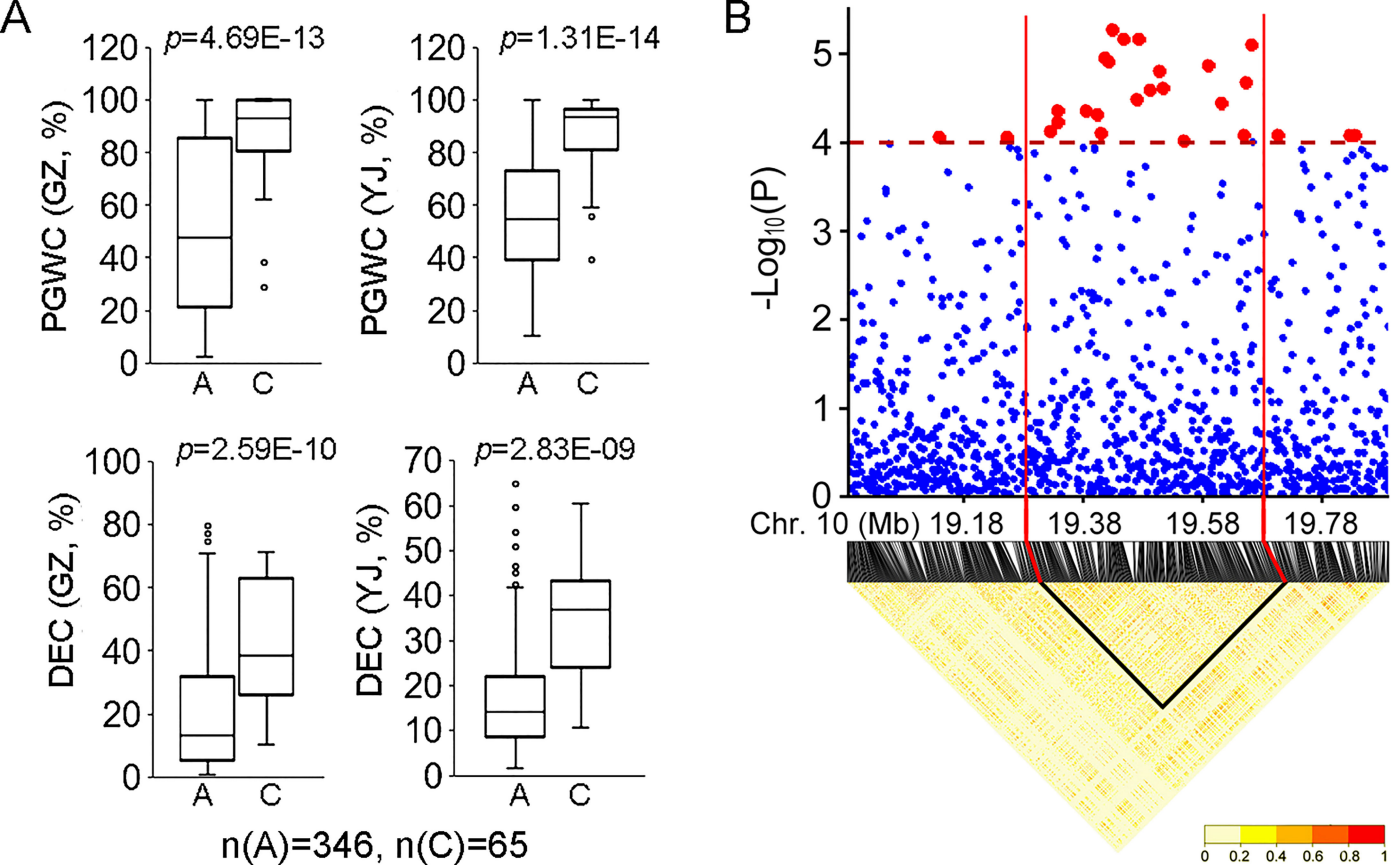
Phenotype and region analysis of *qDEC-10c^1w,2wj^*.**(A)** Phenotypic comparison of PGWC and DEC between accessions with different haplotypes for *qDEC-10c* ^1w,2wj^. Statistical comparison was performed by one-side t-test. **(B)** Local manhattan plot of GWAS and linkage disequilibrium heatmap for *qDEC-10c* ^1w,2wj^.

For the 34 QTLs identified in the present study, *qDEC-10c^1w,2wj^* was co-located with *qPGWC-10^1w,2wj^* and they could be identified in the whole populations in both environments (**Figure 2**). Besides, *qDEC-10c^1w,2wj^* and *qPGWC-10^1w,2wj^* had the most significant *p*-value among the newly identified QTLs (**Table 1**). For *qDEC-10c^1w,2wj^* and *qPGWC-10^1w,2wj^* loci, both PGWC and DEC were significantly different between accessions with different haplotypes (**Figure 3A**). Based on the SNP for GWAS, the LD decay analysis was used to narrow the region of *qDEC-10c* ^1w,2wj^. There was a significant disconnection at 19.28 Mb and 19.68 Mb of chromosome 10 (**Figure 3B**). Because the rice linkage disequilibrium block is about 100-200 kb (Wang et al., 2020). So the *qDEC-10c* ^1w,2wj^ was delimited to an approximately 400 kb region (from 19.28 to 19.68 Mb) on chromosome 10.

### 3.4 Candidate gene analysis of *qDEC-10c^1w,2wj^*

**Figure 4:**
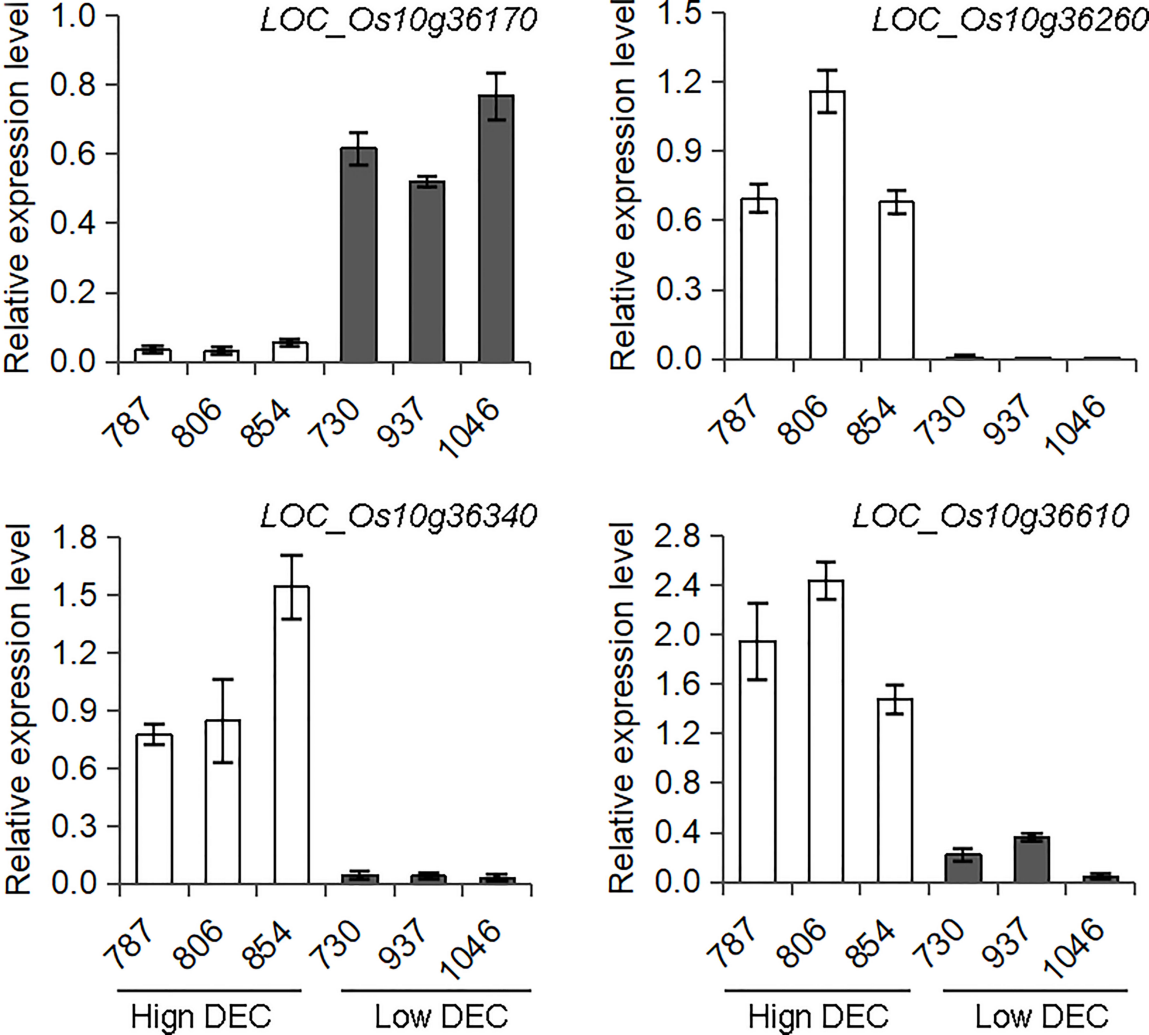
Expression analysis of candidate genes for *qDEC-10c* ^1w,2wj^. The spikes were sampled on the15th day after flowering of three accessions with high DEC (accessions 787, 806 and 854) and three accessions with low DEC (accessions 730, 937 and 1046) were selected based on haplotype analysis to conduct RNA-sequencing and qRT-PCR. Different letters indicate significant difference at *P* < 0.01.

There are 51 annotated genes within the *qDEC-10c* ^1w,2wj^ region (**Table S2**) based on release 7 of the MSU Rice Genome Annotation Project (Kawahara et al., 2013). Since the grain filling stage is the key period for seed development and chalkiness formation, and the differentially expressed genes during this stage may result in variant quality (Yang et al., 2022c; Zhao et al., 2022). To reduce the number of candidate genes, three accessions with high DEC haplotype and three accessions with low DEC haplotype based on haplotype analysis of *qDEC-10c* ^1w,2wj^ were selected for gene differential expression analysis at grain filling stage (15 days after flowering). RNA-sequencing revealed that 28 genes were expressed, of which five genes (*LOC_Os10g36170*, *LOC_Os10g36260*, *LOC_Os10g36270*, *LOC_Os10g36340* and *LOC_Os10g36610*) were differentially expressed between the two sets of contrasting accessions (**Table S2**). qRT-PCR assays confirmed that the expression patterns of *LOC_Os10g36170*, *LOC_Os10g36260*, *LOC_Os10g36340* and *LOC_Os10g36610* were consistent with the RNA-sequencing result (**Figure 4**), while *LOC_Os10g36270* showed inconsistent differential expression compared with the RNA-sequencing results (**Figure S2**). The expression level of *LOC_Os10g36170* in the accessions with high DEC was lower than that in accessions with low DEC (*p* < 0.01), while expression levels of *LOC_Os10g36260*, *LOC_Os10g36340* and *LOC_Os10g36610* in the accessions with high DEC was higher than that in accessions with low DEC (*p* < 0.01) (**Figure 4**).

### 3.5 Phenotype comparisons among different haplotypes of four candidate genes

**Figure 5:**
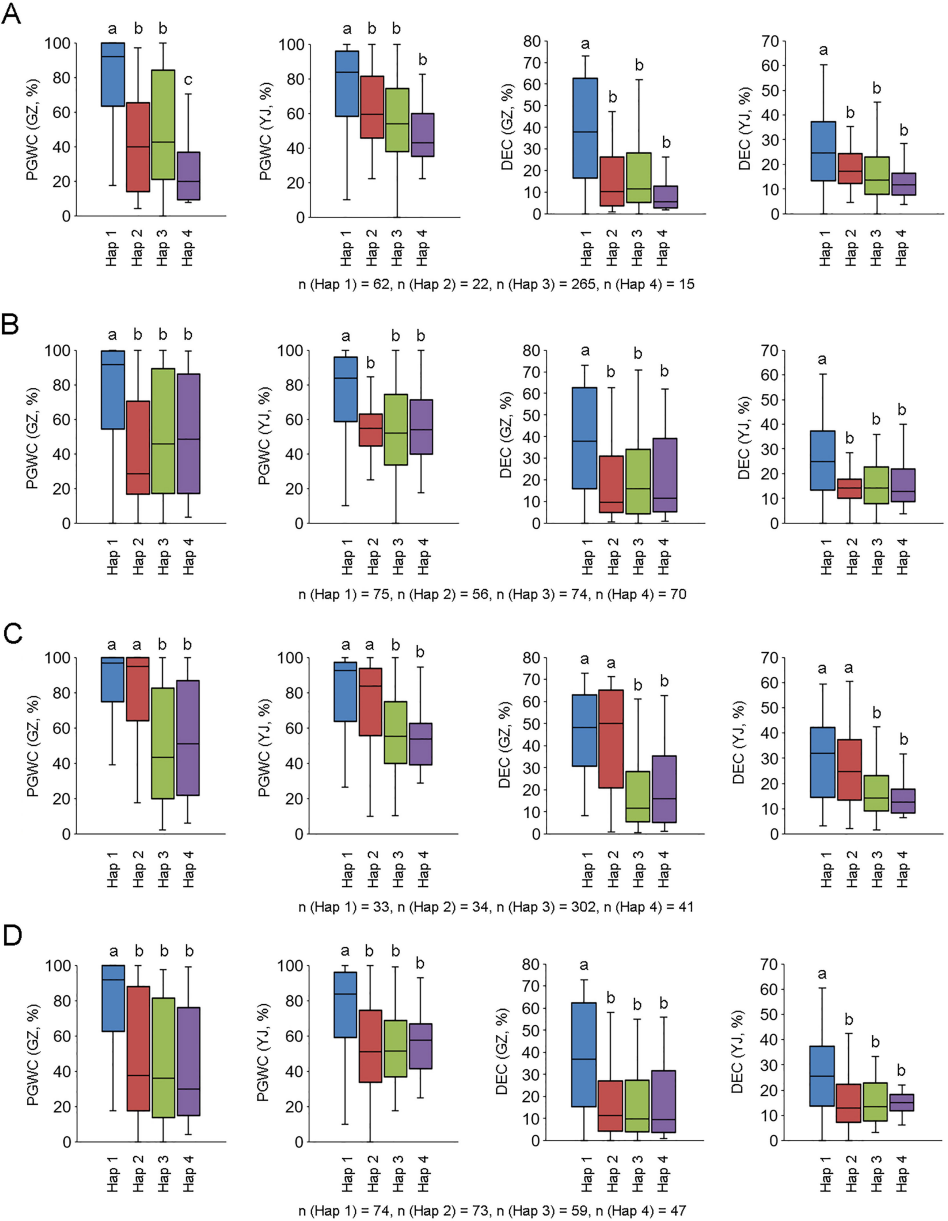
Phenotype comparisons among different haplotypes of four candidate genes. **(A–D)** are *LOC_Os10g36170*, *LOC_Os10g36260*, *LOC_Os10g36340* and *LOC_Os10g36610*, respectively. Different letters indicate significant difference at *P* < 0.01.

We further analyzed the promoter region (2 kb from ATG) of the four candidate genes using re-sequencing data of the 450 accessions, and conducted integration analysis between the four major haplotypes (Hap 1 to 4) of each gene and phenotypes. For *LOC_Os10g36170*, accessions with Hap 1 had higher PGWC and DEC than other haplotypes, and three sequence variations in the promoter region may be the difference between Hap 1 and the other haplotypes (**Figure 5A**; **Table S3**). For *LOC_Os10g36260*, accessions with Hap 1 had higher PGWC and DEC than other haplotypes, and 11 sequence variations in the promoter region may be the difference between Hap 1 and the other haplotypes (**Figure 5B**; **Table S3**). For *LOC_Os10g36340*, accessions with Hap 1 and Hap 2 had higher PGWC and DEC than Hap 3 and Hap 4, and the sequence deletion or existence in the promoter region may account for their differences (**Figure 5C**; **Table S3**). For *LOC_Os10g36610*, accessions with Hap 1 had higher PGWC and DEC than the other haplotypes, but no possible differences were found in the sequence comparison of their promoter regions (**Figure 5D**, **Table S3**).

## 4 Discussion

PGWC and DEC are two important indexes to evaluate rice appearance quality, and some previous studies have reported that there is a high correlation between them (Wang et al., 2017; Qiu et al., 2021). In the present study, the correlation between PGWC and DEC was high (**Figure 1B**). The co-located QTLs identified for PGWC and DEC further confirmed this (**Figure 2**). Therefore, PGWC and DEC could be simultaneously improved by molecular breeding without fear of genetic drag. However, 18 out of 34 QTLs were only associated with one trait (**Figure 2**), indicating that there were different regulatory mechanisms among the two traits. Research on the specific regulation mechanism between PGWC and DEC will further enrich the genetic mechanism of grain chalkiness.

Among the 34 QTLs identified in this study, 11 QTLs were co-localized with the reported QTLs or genes (**Table 1**), indicating the reliability of GWAS analysis and diversity of rice accessions used in this study. Among them, *qPGWC-3b* ^1wj,2wj^ and *qDEC-3* ^2w^ were co-located with *GS3*, and *qPGWC-5a* ^1wij,2wij^ and *qDEC-5a* ^1wij,2wij^ were co-located with *GW5* (**Table 1**). In several studies, *GS3* and *GW5* loci have been reported to control grain shape and chalkiness simultaneously (Qiu et al., 2015; Wang et al., 2017), indicating that the two genes have multiple effects. However, the regulation intensity and direction of different alleles of *GS3* and *GW5* on multiple quality traits are worthy of careful study, which can provide support for the cooperative improvement of grain shape and chalkiness.

In addition to the four QTLs mentioned above, the other seven QTLs were co-located with QTLs that had not yet been cloned. *qDCE-4^1w,2w^* could be identified in the two environments (**Figure 2**) and co-located with *PGC4.4* (Misra et al., 2021)and *qWCR4* (Yun et al., 2016). Misra et al. demonstrated that *PGC4.4* is an important locus affecting chalkiness, and *LOC_Os04g52230* and *LOC_Os04g52240* may be the optimal candidate genes for this QTL by haplotype analysis (Misra et al., 2021). Other QTLs are co-located with some stable or unstable QTLs (**Table 1**). These information suggest that the stable expression of some QTLs is relative, greatly influenced by the environment, and may also be related to the background of research materials.

Because PGWC and DEC are highly positively correlated, it is possible to identify QTLs of PGWC and DEC in the similar region by using the same materials and environments. Wang et al. detected four QTLs affecting DEC and four QTLs affecting PGWC. Among them, *qDEC3* is co-located with *qPGWC3* and *qDEC8* is co-located with *qPGWC8* (Wang et al., 2017). Among the newly identified QTLs in this study, *qPGWC-10* ^1w,2wj^ was co-located with *qDEC-10c* ^1w,2wj^ on chromosome 10, and they were stably detected in both environments and had the most significant *p*-value (**Table 1**). Interestingly, a QTL controlling grain width (*qGW10.2*) (Wang et al., 2017) was also located in the *qDEC-10c^1w,2wj^* region. Since there is a high positive correlation between grain width and chalkiness (Qiu et al., 2015; Wang et al., 2017), whether *qDEC-10c^1w,2wj^* regulates both grain width and chalkiness needs further investigation. We further mined the candidate genes for *qDEC-10c^1w,2wj^*. By integrated analysis of interval definition, differential gene expression and haplotype comparison, four candidate genes were identified for *qDEC-10c^1w,2wj^*. Three genes (*LOC_Os10g36260*, *LOC_Os10g36340*, *LOC_Os10g36610*) encoding unknown functional proteins and one gene (*LOC_Os10g36170*) encoding a lipid transfer protein (LTP) were considered as its candidate genes. LTPs play diverse roles in various biological processes, such as pollen development, stress response, plant signaling and seed maturation (Zhang et al., 2010; Wang et al., 2012). A previous study indicated that down-regulated expression of *OsLTPL36* leads to chalky endosperm and impaired seed germination in rice (Wang et al., 2015). Our results also exhibited that the expression level of *LOC_Os10g36170* in the accessions with high DEC was significantly lower than that in accessions with low DEC (**Figure 4**), which speculates that *LOC_Os10g36170* may functions on regulating the formation of grain chalkiness. In addition, we found some possible key sequence variants related to the expression and phenotype in the promoter regions of three genes by integrating phenotype, haplotype and sequence variation analysis (**Table S3** and **Figure 5**). Further studies are needed to confirm the functions of these candidate genes in grain chalkiness through gain or loss-of function analysis. The identification of novel QTLs and candidate genes for rice appearance quality provides new sources for molecular breeding and cloning of genes associated with rice quality.

## 5 Conclusion

A total of 34 QTLs for grain chalkiness were identified by GWAS in 450 rice accessions. Based on the haplotype analysis, LD decay analysis, RNA-sequencing and promoter sequences analysis, four genes were considered as the candidate genes underlying the potential QTL (*qDEC-10c^1w,2w^*). These results will enrich the genetic bases of grain chalkiness and contributes to molecular breeding for high-quality rice.

## Data availability statement

The original contributions presented in the study are publicly available. This data can be found here: https://www.ncbi.nlm.nih.gov/bioproject/PRJNA948558.

## Author contributions

SZ, WY and JZ designed and supervised this work. XH, JW analyzed the data and wrote the article. LC, HF, TY and JD investigated the phenotype, and performed sample detection. YM and LZ performed the images. JC, DL and BL participated in the revision of the manuscript. All authors contributed to the article and approved the submitted version.

## Supplementary material

Supplementary Figure 1 Manhattan plots of GWAS for PGWC and DEC using different populations in two environments.

Supplementary Figure 2 The expression level of *LOC_Os10g36270* measured by qRT-PCR.

Supplementary Table 1 Information of the 450 rice accessions used in this study.

Supplementary Table 2 The read count of genes within *qDEC-10c* ^1w,2wj^ region measured by RNA-seq.

Supplementary Table 3 Sequence variations in promoter regions of the four candidate genes for *qDEC-10c^1w,2wj^*.

Supplementary Table 4 Primers used in this study.
