# Supplementary figures and images for: Genome-wide association mapping and gene expression analysis reveal candidate genes for grain chalkiness in rice
Source: Front Plant Sci. 2023 Apr 14;14:1184276. doi: 10.3389/fpls.2023.1184276 (PMC10140506; doi:10.3389/fpls.2023.1184276)

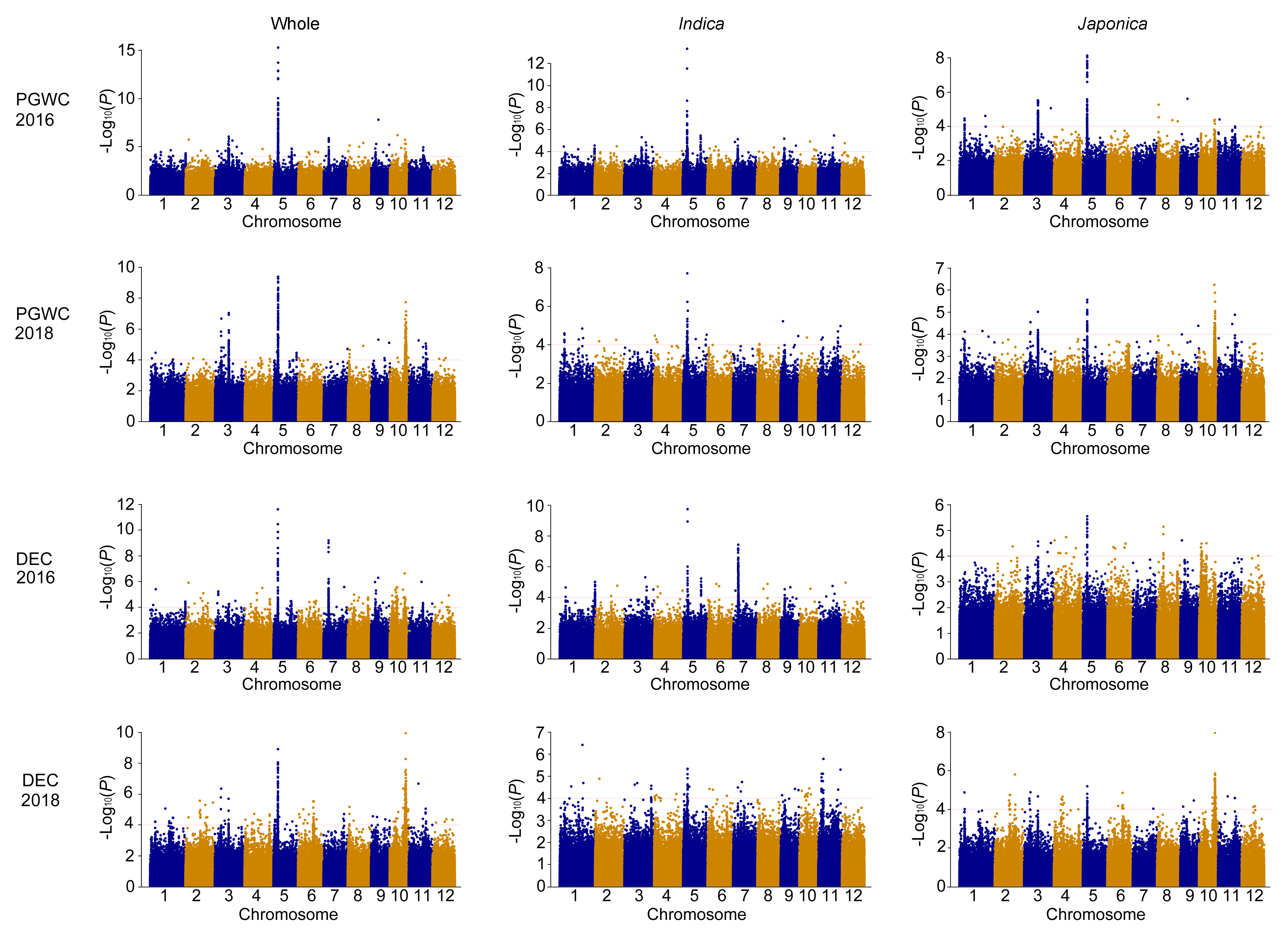

Supplement: Supplementary Figure 1 — Manhattan plots of GWAS for PGWC and DEC using different populations in two environments. [file Image_1.jpeg]

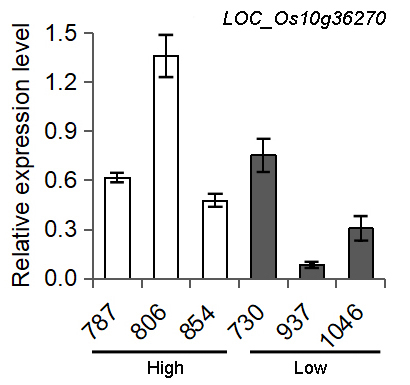

Supplement: Supplementary Figure 2 — The expression level of LOC_Os10g36270 measured by qRT-PCR. [file Image_2.jpeg]
